# Potential Relevance of Bioactive Peptides in Sports Nutrition

**DOI:** 10.3390/nu13113997

**Published:** 2021-11-10

**Authors:** Daniel König, Jan Kohl, Simon Jerger, Christoph Centner

**Affiliations:** 1 Centre for Sports Science and University Sports, Institute for Nutrition, Exercise and Health, University of Vienna, Auf der Schmelz, 61150 Vienna, Austria; 2 Department for Nutritional Science, Institute for Nutrition, Exercise and Health, University of Vienna, 61150 Vienna, Austria; 3 Department of Sport and Sport Science, University of Freiburg, 79102 Freiburg, Germany; jan.kohl@sport.uni-freiburg.de (J.K.); simon.jerger@sport.uni-freiburg.de (S.J.); christoph.centner@sport.uni-freiburg.de (C.C.); 4 Praxisklinik Rennbahn, CH-4132 Muttenz, Switzerland

**Keywords:** muscular performance, connective tissue, muscle recovery, body composition, endurance

## Abstract

Bioactive peptides are physiologically active peptides mostly derived from proteins following gastrointestinal digestion, fermentation or hydrolysis by proteolytic enzymes. It has been shown that bioactive peptides can be resorbed in their intact form and have repeatedly been shown to have a positive effect on health-related parameters such as hypertension, dyslipoproteinemia, inflammation and oxidative stress. In recent years, there has been increasing evidence that biologically active peptides could also play an important role in sports nutrition. Current studies have shown that bioactive peptides could have a positive impact on changes in body composition and muscular performance, reduce muscle damage following exercise and induce beneficial adaptions within the connective tissue. In the following overview, potential mechanisms as well as possible limitations regarding the sports-related effect of bioactive peptides and their potential mechanisms are presented and discussed. In addition, practical applications will be discussed on how bioactive peptides can be integrated into a nutritional approach in sports to enhance athletic performance as well as prevent injuries and improve the rehabilitation process.

## 1. Introduction

The importance of a sports specific diet that is tailored to the type of sports and the associated energy requirements during exercise is largely undisputed. Dietary management helps to maintain an optimal health status of athletes during training and competition but can also prevent injuries, enhance performance and improve regeneration [1,2]. The increasing knowledge about the importance of sport specific adaptations allows individual nutrition recommendations depending on the requirement profile. Robust evidence in the field of sports nutrition is found, e.g., carbohydrates in endurance sports and proteins in strength sports [1,2,3]. In addition to the amount and quality of macronutrients, aspects such as timing and periodization of proteins and carbohydrates are increasingly being researched [4,5,6,7]. One aspect of maximizing physical performance relative to body weight is improving body composition. Body composition plays a very important role in many types of sports, such as endurance sports, aesthetic sports and disciplines with weight classes [8]. It is well known that diet, together with an adequate physical stimulus, is an important component in promoting changes in body composition, such as increasing muscle mass or decreasing fat mass [9]. Nutritive components may improve the metabolism, structure and function of muscles and connective tissue via various signaling pathways [10]. The importance of the connective tissues is increasingly being recognized as structural adaptations within connective tissues, particularly in the area of myotendinous junctions, can help to improve performance and prevent injuries [11].

In general, the effects of proteins of different sources, amino acid compositions and individual amino acids have been the focus of sports specific research for many years. More recently, also studies related to bioactive peptides have yielded interesting and promising results. Bioactive peptides are physiologically active molecules mostly derived from proteins following hydrolysis. The effects of bioactive peptides appear to be due to random interactions with receptors or endogenous proteins such as mammalian target of rapamycin (mTOR), glycogen synthase or glucose transporter type 4 (GLUT-4) that exceed the effects of individual amino acids [12,13,14,15]. Bioactive peptides have repeatedly been shown to have a beneficial influence on health-related parameters such as hypertension, dyslipoproteinemia, inflammation and oxidative stress. In recent years, increasing evidence suggests that biologically active peptides could also play an important role in sports nutrition [16]. Several studies have already shown positive effects after administration of bioactive peptides on relevant, sports-specific aspects such as performance, regeneration and structural adaptations [16]. Currently, much of the existing knowledge is based on cell culture and animal studies but also on human interventional studies, e.g., with hydrolyzed protein. 

The findings on bioactive peptides and their synergistic effect in combination with physical exercise can be beneficial not only for high-performance athletes. Strategies from sport and nutrition could also help to maintain and improve health and well-being for the general population. In this context, the aim is to preserve or even improve relevant aspects related to aging such as muscle mass, functional capacity, mitochondrial function or adaptations within connective tissues [17,18,19,20,21]. Therefore, bioactive peptides could represent another potentially specific and promising strategy to address different aspects of aging. 

In the following sections, studies related to the effects of bioactive peptides on sports specific outcomes will be presented. These sports specific outcomes will be subdivided into results related to body composition, endurance performance and regeneration following muscle damage. 

## 2. Methods

The current literature search was completed at the following electronic databases: Pubmed, ScienceDirect, Scopus and Web of Science. The literature review was performed from articles published up to February 2021. The search string contained the following two segments: the first segment encompassed synonyms of peptides and the second segment included synonyms related to sport performance. All segments were combined using the Boolean operator “AND” and all synonyms within each segment connected with “OR”. The respective MeSHterms were used for each keyword. Due to the limited studies available, both in vitro and in vivo studies were considered relevant.

## 3. Effect on Body Composition

An optimal composition of muscle and fat mass is crucial for many athletes in various sports. It is now well established that nutritional stimuli (e.g., protein intake and amino acid composition), beyond just caloric intake, can support improvements in body composition in terms of an increase in muscle mass and a decrease in fat mass [9,22]. Muscular hypertrophy is particularly important for athletes in the context of increasing muscular strength. Evidence that protein supplementation promotes fat-free mass and muscular strength has been shown in a recent meta-analysis by Morton et al. [23]. Regarding the effects of bioactive peptides, an increase in fat-free mass was observed in non-athletic populations when collagen peptides compared to placebo were taken in combination with 12 weeks of resistance training in young men [24,25], elderly sarcopenic men [26] and premenopausal women [27]. The positive influence of bioactive peptides on muscular hypertrophy could also lead to effects on muscular strength [16]. Benefits on muscle strength were observed in studies with collagen peptides in relation to hand strength in older women [27] and isokinetic quadriceps strength in older men compared to placebo [26]. In other studies with collagen peptides, a descriptive increase in muscle strength were observed in younger as well as older men after 8 and 12 weeks of resistance training compared to placebo, however, these improvements were not statistically significant [24,25,28].

With respect to muscle mass, an upregulation of anabolic signaling is necessary to induce muscular hypertrophy [29]. The stimulation of the mTOR signaling pathway via amino acids such as leucine is one mechanism known to increase muscular protein synthesis [30,31]. In addition, small peptides such as the dipeptide hydroxyproline-glycine have also been shown to activate this signaling pathway in vitro, thereby enhancing hypertrophy in myotubes [32]. Since dipeptides could also be detected in the blood and thus seem to be bioavailable after ingestion of collagen peptides [33], the effect of collagen peptides on body composition is an interesting field of research. Remarkably, collagen peptides have an incomplete amino acid spectrum and low leucine content. Therefore, one might expect a small anabolic effect [34]. However, the positive effect of collagen peptides on body composition could possibly be explained by the signaling effect of bioactive peptides. 

Besides collagen peptides, there are many peptides from other sources that would also be potential candidates. While the effects of whey protein on lean mass are well described [35], bioactive peptides in whey hydrolysate have received little attention. Besides the high biological value and high content of leucine [31,36,37,38], the bioactive peptides in whey hydrolysate could play a role in the observed anabolic effects. Based on the observations in rats that the dipeptide leucine-valine increased the expression of mTOR, it may well be that dipeptides in whey hydrolysate have similar anabolic effects to leucine as a single amino acid [12]. The extent of the influence of bioactive peptides in whey hydrolysate is difficult to determine; two studies found no benefits from supplementation in combination with a resistance training of whey hydrolysate compared to whey protein in young men [39,40].

## 4. Effects on Endurance Performance

Apart from changes in body composition and biomechanical mechanisms, metabolic factors such as improvements in fat oxidation, glucose availability and consumption, as well as muscle glycogen replenishment, play a crucial role in endurance performance [41]. Whether or how bioactive peptides or proteins can enhance these effects is not well understood and their influence on endurance performance is still controversial [16].

To date, few studies have investigated the influence of protein hydrolysates or small peptides on endurance performance. Most recently, it has been shown that collagen peptide supplementation in women can support the effects on endurance performance during concurrent training. A total of 12 weeks of concurrent training resulted in a significant improvement in running distance in a one hour time trial compared to the placebo group. This improvement could not only be explained by metabolic improvements. The significant increase in fat-free mass and strength observed in subjects receiving the collagen peptides [42] may also suggest that biomechanical improvements, leading to enhanced muscle work efficiency, could also be involved in enhancing endurance performance. In another study, Hansen and colleagues observed a performance benefit from supplementation with whey hydrolysate plus carbohydrates compared to carbohydrates alone during a one-week training camp [43]. A performance enhancing effect could also be observed with casein hydrolysate in combination with carbohydrates compared to carbohydrates alone. The improvement in performance occurred in the last third of the 60 km cycling time trial [44]. This could potentially be explained by the results of Oosthuyse et al. [45], who found an increase in fat oxidation following casein ingestion. This effect could lead to a saving of carbohydrate stores and had a positive effect on performance in the time trial compared to the placebo. In the study, performance was not significantly improved by carbohydrate alone or carbohydrate plus whey hydrolysate compared to placebo [45].

Possible explanations for an improved endurance performance include an increased glycogen uptake and storage in muscle cells. For example, isoleucine-valine, a peptide from hydrolyzed whey protein, has been shown to stimulate glucose uptake in vitro [13] and to increase muscle glycogen content after exercise in rats [12]. Observations in mice could show that hydrolyzed whey protein leads to an activation of glycogen synthase [14]. In rats, an increased translocation of GLUT-4 transporters from the cytoplasm into the cell membrane in an insulin-independent manner was demonstrated [15]. In addition, it was also shown that whey hydrolysate further increased the uptake of glucose into skeletal muscle cells compared to whey protein [14,15]. In summary, in rodents, whey hydrolysate in combination with glucose appears to raise skeletal glycogen stores more than glucose and whey protein or glucose alone [46]. However, this effect could not always be confirmed in human studies. Thus, in humans, the administration of hydrolysates from casein, whey or wheat plus carbohydrates showed no advantages with regard to carbohydrate uptake or metabolism compared to a comparable dose of carbohydrates [47,48,49].

Another way in which athletic performance can be optimized by nutrition is by influencing endothelial function. In this context, nitrate supplementation is already used by some athletes, but also other food components such as omega-3 fatty acids or some secondary plant products also have a beneficial effect on endothelial function, thus improving muscle perfusion [50,51,52]. Bioactive peptides from various sources, such as whey [53,54,55,56], plants [57] or eggs [58], could be a promising research subject for acute effects on performance during high intense or prolonged exercise. It is already known that peptides from these sources can improve endothelial function by suppressing the angiotensin converting enzyme (ACE). Thus, it is possible that specific bioactive peptides can be used to acutely influence performance. However, there are still many open questions and, therefore, the influence of different bioactive peptides on biomechanical and metabolic factors following endurance or concurrent training needs to be investigated in further studies.

## 5. Effects on Muscle Damage

Performing unaccustomed exercise has repeatedly been shown to result in exercise-induced skeletal muscle damage (EIMD) [59,60]. Especially eccentric muscle work (e.g., the lengthening of a contracting muscle) seems to facilitate structural damage including Z disk streaming and disrupted sarcomeres, and thus impairments within the excitation-contraction coupling system [61,62]. Previous findings demonstrated that besides the involvement of muscle tissue, connective tissue can also be affected by excessive mechanical loading [63,64]. As a consequence, this may lead to extracellular matrix (ECM) disruption [63,64]. 

Signs of EIMD are temporary but typically persist for up to several days after cessation of exercise. Frequently reported consequences include muscle soreness, decrements in force-generating capacity as well as elevated levels of specific biomarkers (e.g., creatine kinase (CK), lactate dehydrogenase or myoglobin) [65]. The fact that severe EIMD might compromise athletic performance for several days [66,67] highlights the need for new strategies which could help to attenuate EIMD [68]. Besides approaches in the field of pharmacological and physical therapy [69], nutritional strategies, especially the intake of bioactive peptides [70], have recently gained increasing attention [68]. 

Until now, several investigations have been conducted with peptides and hydrolysates on muscular performance following exercise to volitional fatigue. In 2010, Buckley and colleagues [71] performed a randomized, placebo-controlled trial and examined the effects of whey hydrolysate on muscle soreness, serum CK activity and TNF-alpha concentrations following unaccustomed eccentric exercise. The results demonstrated that hydrolyzed whey facilitated a rapid regeneration of force capacity, assessed via maximal isometric contractions, which was significantly enhanced compared to the control group [71]. Interestingly, the authors did not report any group differences in muscle soreness and found no evidence for changes in CK or TNF-alpha. In a recent study, Brown and colleagues [72] investigated the effects of whey protein hydrolysate on EIMD and recovery following exhaustive repeated-sprint exercises in females. To assess changes over time, biomarkers of EIMD were assessed up to three days following cessation of exercise. The findings demonstrated that the supplementation with whey hydrolysate led to lower CK levels at 48h and attenuated the decline in muscle function (evidenced by an improved reactive strength index and higher flexibility). These positive short-term effects of hydrolysates are in accordance with the findings from further studies [43,73], although some researchers reported contradictory results [74]. 

Regarding longitudinal trials, Lollo et al. [75] investigated the effects of whey hydrolysate supplementation during a 12-week soccer-specific intervention on performance and recovery. The results supported the findings of acute trials and demonstrated that whey hydrolysate supplementation significantly attenuated markers of muscle damage such as CK (−42% reduction) and lactate dehydrogenase (−30% reduction) compared to control. 

Besides whey peptides, collagen peptides have also been investigated regarding muscular recovery. A previously published randomized-controlled trial by Clifford and co-workers [70] investigated the effects of collagen peptides on muscle function and muscle damage. To quantify muscle function and damage, the authors implemented maximal voluntary contractions as well as countermovement jumps. Muscle soreness was assessed via a visual analogue scale. The results from this trial revealed that explosive force production was more rapidly restored in the collagen peptide group compared to the control group. Additionally, evidence for attenuated muscle soreness following strenuous exercise was observed with the ingestion of collagen peptides. Furthermore, positive effects on an accelerated muscle recovery were also seen following the ingestion of isolated soy protein [76]. 

In general, however, it needs to be mentioned that evidence is rather scarce on whether peptides or protein hydrolysates facilitate muscle recovery and decrease muscle damage following intensive exercise. It should be particularly emphasized that all studies did not directly assess the specific peptide composition in their supplements, which limits the conclusions about the direct effects of bioactive peptides on muscle damage. The fact that some studies report beneficial effects is encouraging but warrants more research with a more specific look on the bioactivity of individual peptides.

### 5.1. Effects on Protein Synthesis

Following high-intensity and strenuous exercise, it is generally accepted that both muscle protein synthesis as well as degradation are increased. When the stimulation of muscle protein synthesis is greater compared to protein breakdown, muscle net balance is increased. This can be further improved by the addition of exogenous amino acids, which provide the induction of an anabolic environment and thus facilitate tissue remodeling [77]. Bioactive peptides are increasingly considered as a crucial factor for regulating muscle protein turnover. In previous in vitro trials, it has been demonstrated that the collagen derived dipeptide hydroxyprolyl-glycine (Hyp-Gly) induces myotube hypertrophy by activating anabolic signaling pathways such as PI3K/Akt/mTOR [32]. Therefore, Hyp-Gly potentially could facilitate an accelerated recovery of the contractile machinery, allowing an accelerated force recovery following EIMD. This evidence, however, relies on findings from in vitro studies and needs to be confirmed by future in vivo trials.

### 5.2. Effects on Inflammation 

Acute exercise-induces muscle trauma initiates a sequence of cellular processes including a robust inflammatory response [78,79]. Depending on the type of exercise, elevated levels of inflammatory markers can be found for up to several days [80,81]. Despite the fact that an adequate level of inflammation is pivotal for adequate wound healing [82] and satellite cell response [83], a chronic pro-inflammatory status impairs muscle regeneration [84] and induce lysis of muscle membranes [80]. Consequently, it might be assumed that specific nutritional requirements might help to maintain an optimal inflammation level within the muscle following EIMD. 

To date, much of the current evidence on the anti-inflammatory potential of bioactive peptides is based on mammalian cell culture experiments [85]. Previous trials that have focused on casein derived peptides found that tripeptides with the sequence Val-Pro-Pro demonstrate a robust anti-inflammatory potential by inhibiting the nuclear factor kappa B pathway [86]. Similarly, Jiehui and colleagues (2014) found that the peptide Gln-Glu-Pro-Val-Leu (also obtained from casein) seemed to be involved in nitric oxide release and augmenting the production of anti-inflammatory cytokines such as IL-4 and IL-10 [87]. A recent animal experiment investigated the effects of a specific dipeptide (L-alanyl-L-glutamine) in male Wistar rats. The findings indicated that this dipeptide induced cytoprotective effects being mediated by heat shock protein-70 (HSP 70) responses. Additionally, plasma levels of CK, TNF-alpha and lactate dehydrogenase were decreased which might be associated with enhanced muscle cell protection and attenuated inflammation following exercise [88]. Despite the evidence in animals, research in humans is still scarce and it is unknown whether these anti-inflammatory effects in in vitro and animal experiments can contribute to a better muscle recovery after EIMD in humans.

### 5.3. Effects on Oxidative Stress

Previous research has shown that several peptides demonstrate a high antioxidative capacity. Without adequate clearance of reactive oxygen species (ROS) by antioxidants, ROS are capable of damaging virtually all major cellular constituents [89]. Although physiological levels of ROS play an important role as regulatory mediators in intramuscular signaling processes [90], disproportionate amounts have been discussed to be involved in prolonged inflammation and exaggerated muscle damage following exercise [91]. 

In terms of bioactive peptides, previous trials have again primarily focused on peptides derived from milk products. In a study by Suetsuna and colleagues (2000), casein peptides with the sequence Tyr-Phe-Tyr-Pro-Glu-Leu have been demonstrated to have free radical scavenging effects [92]. Within their study, the authors sequentially removed amino acids from the peptide and found that the highest antioxidant capacity can be achieved with the dipeptide Glu-Leu [92]. Also with casein, Kitts [93] has shown that phosphopeptides are able to demonstrate free radical quenching activity. 

Similar to casein peptides, several other peptides have shown to possess antioxidant potential [94] including whey [95], collagen [96] or soy [97,98]. Nevertheless, it is currently not known exactly to what extent this antioxidant capacity contributes to changes in EIMD in humans. 

In summary, previous findings revealed that various peptides demonstrate distinct effects on muscle damage via optimizing the inflammatory response, myofibrillar re-structuring or minimizing oxidative stress. Nevertheless, current evidence is still far from being conclusive and further in vivo trials in humans are necessary to elucidate potential benefits of bioactive peptides on muscle damage. 

## 6. Effects on Connective Tissue

Connective tissue is essential for the execution of human movements and therefore, the properties and the function of connective tissue is an important determinant for athletic performance [99,100]. Intense and/or unaccustomed prolonged physical exercise induces enhanced stress and strain which could result in tendinopathy and functional joint pains among athletes [101]. Connective tissue needs to adapt its structure and composition permanently to the physical requirements, in order to optimally support athletic performance, to cope with enhanced loading and to prevent degeneration [102]. Exercise-induced adaptions as well as nutrition seems to influence connective tissue. More specifically, its alterations of material and morphological adaptions after supplementation of biologically active peptides have gained increasing attention in the scientific community [103].

The structure of its extracellular matrix (ECM) and specifically its protein composition defines mechanical properties of connective tissue [104]. Since collagen is the most abundant matrix molecule [102,104] research of connective tissue adaptions has focused on its synthesis in fibroblasts [105].

### 6.1. Effects on Tendon Properties

The ordered assembly in fibrils contributes to the characteristic elastic behavior of tendons and allows efficient storing and returning of energy during tensile loads [106]. Reinforcement of the ECM by an elevated synthesis rate in tenocytes increases its ability to tolerate higher stress levels [107]. Intact and healthy tendons improve athletic performances such as running economy [100] and decrease the risk of injury [107].

To date, multiple trials have investigated the stimulating effects of bioactive peptides on collagen synthesis in tendons. Schunck and Oesser (2013) reported a pronounced stimulatory effect on RNA expression and biosynthesis of matrix molecules after adding collagen hydrolysates to primary fibroblasts in vitro. These findings were confirmed by an in vivo animal study, which revealed that collagen peptides supplemented to rabbits over a time period of 56 days increased collagen fibril diameter and possibly improved mechanical properties of the Achilles tendon [108]. The authors contributed this effect to the high concentration of glycine within the supplement [109,110]. Alongside single amino acids, the authors suspected a promoting effect of the tri-peptide glycine-proline-arginine (Gly-Pro-Arg). It shows various functions in the human body; for instance, antiaggregatory effect has been shown in platelets [111]. Moreover, gelatin supplementation led to increased levels of collagen synthesis markers in blood after rope skipping exercises [112]. Since gelatin per se does not contain collagen peptides, conclusions from these results on biologically active peptides remain speculative. However, their formation during digestive degradation processes could potentially contribute to the stimulating effect.

On the one hand, there is not enough evidence highlighting the effects of a supplementation with collagen peptides on tendon adaptions in humans, due to a lack of studies in vivo. On the other hand, a beneficial influence of collagen peptide supplementation is conceivable, since a stimulating effect of collagen peptides on tendon fibroblasts is indicated in vitro [113] and it shows a promoting effect on tendon adaptions in animals [108] as well as increased collagen synthesis after gelatin intake in humans [112]. Further research is needed for practical recommendations. 

Supplementation with proteins containing high concentrations of leucine and other essential amino acids (EAA) is currently a major field of myofibrillar protein synthesis research [23]. Its impact on tendinous adaptions has also gained interest. In an animal study, Barbosa and colleagues (2012) revealed a stimulating effect of a leucine-rich diet on collagen content and biomechanical adaptions of the deep digital flexor tendon in malnourished rats conducting aerobic physical exercise [114]. In humans, a 12-weeks high-intensity resistance training in combination with supplementation of high-leucine whey hydrolysate led to an augmented cross-sectional area of the proximal patellar tendon [115]. This might demonstrate an advantageous impact of a supplementation with EAA rich peptides on exercise induced adaptions in healthy tendons. It has to be mentioned that no specific peptides have been identified in these studies.

Due to the pronounced and repetitive loading of tendons in sports, tendinopathy is a severe issue among athletes [101]. Among other therapies, peptide intake was subject of research about tendon healing. The results of a study by Praet and colleagues (2019) indicate a beneficial effect of exercise combined with collagen hydrolysate supplementation on tendon healing in subjects with Achilles’ tendinopathy. The authors attribute it to the high glycine uptake of 1.1g per day. Glycine was related to improved matrix organization strength and tenocyte remodeling by modulation of TNF-alpha, matrix metalloproteases and collagen precursors [116,117]. In Achilles tendinopathic patients with extracorporeal shockwave therapy, a positive influence of a supplement containing arginine and collagen could be demonstrated [118]. These results indicate a beneficial effect of single amino acids and maybe specific peptides on tendon healing therapy [119]. Further research is needed to characterize the role of biologically active peptides in detail and to detect potential mechanisms.

### 6.2. Effects on Cartilage and Functional Joint Pain

Athletes show an increased risk of articular cartilage lesions leading to functional joint pain [120]. One therapeutic attempt includes the treatment and prevention of degenerative articular processes by stimulation of collagen synthesis with biologically active peptides [103].

In vitro, cultured chondrocytes showed stimulation of proteoglycans as well as type II collagen and increased protease activity [121,122]. Several studies showed beneficial effects of collagen peptide supplementation on pain and joint mobility of athletic subjects suffering from functional joint pains, in vivo [123,124]. According to the authors, the stimulatory effect of collagen peptides on ECM-protein synthesis could likely explain these results. Although the exact mechanism remains unclear, specific biologically active signaling peptides derived from hydrolyzed collagen such as hydroxyproline-proline-glycine (Hyp-Pro-Gly) and hydroxyproline-glycine (Hyp-Gly) are suspected to trigger those reactions [32,124,125,126].

Research indicates that a supplementation with whey hydrolysate might stimulate collagen synthesis [114]. Thus, the potential influence of whey hydrolysate supplementation on cartilage regeneration and recovery of functional joint pains should be discussed. However, because of a lack of studies, statements about the effects of a whey hydrolysate supplementation on articular cartilage remain speculative.

## 7. Practical Applications 

The primary goal in competitive sport is to increase and optimize athletic performance. For this purpose, the training process is often intensified in regular phases of the yearly competition cycle, which substantially increases the mechanical and metabolic stress acting on the athlete [127]. In order to optimally prepare for these high-intensity phases, both stringent exercise routines as well as dietary strategies have been incorporated into training management in elite sport [77,128,129]. In particular, certain peptides as well as hydrolyzed proteins have been demonstrated to have beneficial effects with regard to myotendinous adaptations and an accelerated recovery. 

Indeed, previous in vitro and in vivo studies have confirmed that several bioactive peptides elicit strongly upregulating effects on muscle protein synthesis [32] or increase muscular strength [26] and recovery [70]. This is of special interest for sports, where large forces against an object or opponent (e.g., combat sports) are needed. Interestingly, recent discussions in the scientific community address the fact that these peptides may also be helpful for reducing injuries and accelerating return-to-sport [130]. Besides positive effects on strength and recovery, recent investigations found that hydrolyzed proteins might also influence endurance performance and metabolism [42,43,45]. Hansen et al. (2015), for example, revealed that whey hydrolysate in combination with carbohydrate enhanced running performance and facilitated recovery from strenuous training compared to carbohydrate alone [43]. These findings indicate that it might also be advantageous to incorporate nutritional interventions with certain peptides into sports with endurance aspects. In this context, it needs to be mentioned that evidence is still scarce and also contradictory findings exist in the current literature [131]. 

## 8. Conclusions

In summary, various findings point towards potential beneficial effects of specific bioactive peptides on exercise performance as well as recovery and tissue repair. Although these preliminary findings are promising, further studies are warranted before definitive conclusions can be drawn. In particular, studies with specific or known peptide sequences are lacking in order to be able to evaluate the effects of specific peptides. This highlights the need for further research on the modes of action both in vitro and in vivo. Based on the present narrative review, future studies need to focus on (i) the effects of bioactive peptides on biomechanical as well as metabolic responses in humans and (ii) elaborating the underlying physiological mechanisms of various peptides. Lastly, (iii) the investigation of potential effects in different sport populations are necessary in order to evaluate potential field of applications. Moreover, the majority of existing studies examined the effects of hydrolyzed proteins but not of individual bioactive peptide forms. 
